# AlphaMissense for Identifying Pathogenic Missense Mutations in DNA Damage Repair Genes in Cancer

**DOI:** 10.1200/PO-24-00908

**Published:** 2025-06-26

**Authors:** Shu Yazaki, Xin Pei, Simon Powell, Atif Khan, Jeremy Setton, Nadeem Riaz

**Affiliations:** ^1^ Department of Radiation Oncology, Memorial Sloan Kettering Cancer Center, New York, NY

## Abstract

**PURPOSE:**

AlphaMissense is a new artificial intelligence–based approach to predicting the pathogenicity of missense variants. However, whether its predictions can be directly applied to clinical decision making remains unclear. This study aimed to evaluate the accuracy of AlphaMissense predictions for DNA damage repair (DDR) genes using genomic and clinical characteristics.

**METHODS:**

Sequencing data from 56,965 patients with cancer who underwent Memorial Sloan Kettering–Integrated Mutation Profiling of Actionable Cancer Targets (MSK-IMPACT) testing between April 2015 and March 2023 and data from The Cancer Genome Atlas (TCGA)/Pan-Cancer Analysis of Whole Genomes (PCAWG) were analyzed. AlphaMissense pathogenicity was evaluated in six commonly mutated DDR genes. Missense mutations were classified into three categories on the basis of AlphaMissense and OncoKB: known pathogenic, newly identified pathogenic by AlphaMissense, or benign.

**RESULTS:**

In the MSK-IMPACT cohort, 1,182 (17.5%) of 6,743 unique DDR gene missense mutations were newly identified as pathogenic. In breast, ovarian, pancreatic, and prostate cancers, homologous recombination (HR)–deficiency signatures were more common in tumors with new pathogenic missense mutations in *BRCA1/2*, *PALB2*, and *RAD51C* than in benign missense mutations (66.7% *v* 35.2%, *P* = .021). In the TCGA/PCAWG data set, HR-deficiency signatures were also more frequent in tumors with new pathogenic mutations than in wild-type (46.2% *v* 19.2%, *P* = .036). For tumors with *POLE* missense mutations, there were no significant differences in tumor mutation burden and POLE-associated signatures between new pathogenic and benign mutations. Patients with new pathogenic *ATM* missense mutations had fewer *TP53* mutations (30.5% *v* 54.6%, *P* < .001) and showed improved irradiated tumor control (hazard ratio, 0.58 [95% CI, 0.35 to 0.95]; *P* = .03) compared with those with benign missense mutations.

**CONCLUSION:**

Our findings suggest that AlphaMissense can help identify previously unknown pathogenic DDR gene mutations, but its accuracy is gene-dependent. AlphaMissense prediction still requires additional confirmation with clinical and functional validation.

## INTRODUCTION

Genomic instability, a hallmark of cancer that facilitates oncogenesis, is often caused by defects in DNA damage repair (DDR).^1^ Deficiencies in the DDR are frequently caused by germline or somatic mutations in genes in specific pathways including mismatch repair (MMR), homologous recombination (HR), or DNA polymerases. Identifying DDR defects is clinically significant as they can be therapeutically exploited with therapies such as immune checkpoint blockade (ICB) or PARP inhibitors.^2-4^ However, not all mutations in DNA repair genes result in inactivation of a pathway, and although some mutations are known to be pathogenic, the significance of others is unclear (eg, variants of unknown significance [VUS]). Identifying which DNA repair mutations are pathogenic remains a significant clinical challenge with important therapeutic implications.^5^ One approach to elucidate the function of VUS is to use multiplex assays of variant effects (MAVEs), which can measure the functional effects of variants proteome-wide. However, this method is time- and resource-intensive and depends on experimental quality. Recently, artificial intelligence–based approaches using protein structure prediction, such as AlphaMissense, enhance the accuracy of pathogenicity predictions for missense variants.^6^ Although AlphaMissense outperforms previous methods in categorizing ClinVar and MAVE variants, its clinical utility in decision making still requires additional validation.

**CONTEXT**

**Key Objective**
AlphaMissense is a new artificial intelligence–based approach to predicting the pathogenicity of missense variants. However, its utility for clinical decision making has not yet been validated. Accurately interpreting the pathogenicity of missense mutations in DNA damage repair genes is crucial for assessing cancer susceptibility and guiding specific treatments.
**Knowledge Generated**
Using clinical tumor-blood genomic data from patients with solid tumors, we found that AlphaMissense predictions are accurate for *BRCA1/2, PALB2, RAD51C*, and *ATM* but not for *POLE.* These predictions did not correlate with accuracy of protein structure prediction.
**Relevance**
The accuracy of AlphaMissense varies by gene, and its predictions may still need to be validated with additional clinical and functional evidence.

Interestingly, tumors with DNA repair defects not only have specific therapeutic vulnerabilities but also produce characteristic forms of genomic instability that can be detected by analyzing mutational signatures from tumor genomes. For example, *BRCA1/2*-deficient tumors exhibit a characteristic mutational signature (signature 3) indicative of defective HR.^7^ This signature was also associated with loss-of-function (LoF) mutations in other HR-related genes, including *PALB2* and *RAD51C.*^8^ MMR deficiency and *POLE* proofreading deficiency typically lead to a hypermutator phenotype and correlate with improved response to ICB.^9,10^ We recently identified that tumors with LoF mutation in *ATM*, an essential gene in DNA damage detection and repair signaling, are strongly associated with clinical benefit from radiotherapy (RT).^11^ Consequently, identifying pathogenic DDR mutations is crucial to guide therapeutic strategies.

Here, we hypothesized that AlphaMissense classification of VUS could identify new bona fide pathogenic mutations in DDR genes, which could have clinical implications. We sought to use known mutational signatures of defects in DDR genes as well as canonical therapeutic and molecular features of DDR defective tumors to evaluate the accuracy of pathogenicity identified by AlphaMissense.

## METHODS

### Study Cohort

We analyzed clinicogenomic data from 56,965 patients with advanced cancer who underwent targeted panel sequencing using Memorial Sloan Kettering–Integrated Mutation Profiling of Actionable Cancer Targets (MSK-IMPACT) from April 2015 to March 2023 (Data Supplement, Fig S1). This study was approved by the Memorial Sloan Kettering Cancer Center (MSKCC) Institutional Review Board. All patients provided written informed consent to an institutional prospective sequencing protocol.

### Mutation Analysis

MSK-IMPACT is a hybridization capture–based next-generation sequencing assay analyzing tumor and matched normal blood for all protein-coding exons of 341-505 cancer genes, as previously described.^12^ Germline variant calling was performed using MuTect and Genome Analysis Toolkit Haplotype caller. Allele-specific copy-number analysis was performed using FACETS.^13^ Tumor mutation burden (TMB) was determined by normalizing the number of nonsynonymous alterations on the basis of the sequenced genome.^14^ Microsatellite (MS) status was determined by MSIsensor, considering MS instability-high with a score ≥10, MSI-intermediate with a score from ≥3 to <10, and MS-stable with a score <3.^15,16^

### Classification of Missense Mutations

We selected six DDR genes to evaluate the pathogenicity predictions of AlphaMissense for missense mutations. *ATM*, *BRCA1/2*, and *POLE* were the most commonly mutated DDR genes and had therapeutic vulnerabilities to specific treatments. *PALB2* and *RAD51C* were included in the analysis as core-HR genes with *BRCA1/2* as their genetic defects are associated with HR-deficiency signatures.^17,18^ AlphaMissense classifies mutations as either likely benign, likely pathogenic, or ambiguous.^6^ We excluded missense mutations with ambiguous predictions from further classification. Missense mutations classified as pathogenic by AlphaMissense, but previously known to be pathogenic in the literature or classified as oncogenic or likely oncogenic by OncoKB^19^ were classified as known pathogenic. Mutations newly identified as pathogenic by AlphaMissense were classified as new pathogenic. The remaining mutations were classified as benign. Hence, mutations were classified into three categories: known pathogenic, new pathogenic, and benign.

### Mutational Signature Analysis

Mutational signature analysis was performed using the R package SigMA.^20^ Only samples with ≥5 signle nucleotide variants (SNVs) were analyzed. Predictions were made separately using the corresponding tumor type settings when available; otherwise, the *tumor_type* parameter was set to other. We obtained mutational signature exposure of HR deficiency (signature 3, referred to as Sig3) and POLE-proofreading deficiency (signature 10a +10b) calculated by non-negative least squares. Signature exposures were classified using a 20% cutoff (>20% *v* ≤20%) on the basis of the lowest possible mutational signature that can be detected with sufficient confidence in samples with ≥5 SNVs.

### MSK-IMPACT Cohort of BOPP Cancers

To examine the association between the pathogenicity of core-HR gene mutations and mutational signatures, genomic data from patients with four tumor types associated with HR deficiency (breast, ovarian, prostate, and pancreatic cancers; referred to as breast, ovary, pancreatic, and prostate [BOPP] cancers) were analyzed. LoF mutations in core-HR genes were defined as those causing truncation, frameshift, or a deleterious splice site and classified as known pathogenic mutations. Biallelic mutations were defined as germline or somatic mutations with loss of heterozygosity (LOH) of the wild-type allele. Patients with unknown allelic status of core-HR genes and those with monoallelic core-HR mutations were excluded.

### MSK-IMPACT Cohort of Cancers With *POLE* Missense Mutations

We obtained genomic data from patients harboring *POLE* missense mutations to investigate the association between the pathogenicity of *POLE* mutations, TMB, and mutational signatures. The exonuclease domain (ED) was defined as ranging from amino acid 268 to 471. Cancer types representing <1% of the total samples (<20 samples) were excluded. Patients without TMB and MSIsensor scores were also excluded. To clarify the independent effect of *POLE* VUS on TMB and mutation signature, patients with MSI-intermediate and MSI-high were also excluded.

### MSK-IMPACT Cohort of Cancers With *ATM* Missense Mutations

First, we obtained genomic data from patients with *ATM* missense mutations to examine the association between the pathogenicity of *ATM* mutations, mutation location, and comutations in *TP53*. FRAP, ATM and TRRAP (FAT)/kinase domain was defined as ranging from amino acid 1,960 to 3056. Next, we analyzed patients with *ATM* missense mutations who received RT at MSKCC. Details of the cohort were previously described.^11^ The incidence of irradiated tumor progression was compared between tumors with new pathogenic *ATM* missense mutations and those with benign missense mutations. Irradiated tumor progression was defined as the radiographic or pathologically confirmed progression and/or relapse within the RT planning target volume. The multivariable analysis was performed by adjusting cancer types, age, *TP53* mutations, gross disease, metastatic status, biological effect dose, and RT sites.

### TCGA and PCAWG Analysis

To further validate the performance of AlphaMissense prediction for core-HR gene mutations, we also analyzed publicly available whole-exome sequencing/whole-genome sequencing (WES/WGS) data including The Cancer Genome Atlas (TCGA, WES, n = 8,178) and Pan-Cancer Analysis of Whole Genomes (PCAWG, WGS, n = 1,484).^21^ We extracted patients with BOPP cancers from the two data sets and created a TCGA/PCAWG cohort as a validation cohort. Further details were described in the Data Supplement.

### Protein 3D Structures

The protein structures predicted by AlphaFold were obtained from AlphaFoldDB.^22^ We evaluated the local confidence of AlphaFold2 structure prediction using the predicted local distance difference test (pLDDT). Pairwise alignments between AlphaFold2 structure predictions and experimentally derived protein structure were performed using the RCSB.org^23^ alignment tool.^24^ The similarity between AlphaFold2 and experimental structures was assessed using root-mean-square deviation (RMSD).

### Statistical Analysis

Categorical variables were compared by the χ^2^ test or Fisher exact test. Comparisons of the proportion of mutational signatures between different groups were analyzed with the Wilcoxon rank-sum test. A Fine-Gray regression model was used to evaluate the cumulative incidence of irradiated tumor progression and the influence of covariates, treating death as a competing risk. Statistical analyses were performed using R software version 4.3.2 (R Project for Statistical Computing, Viena, Austria). All significance tests were two-tailed, and *P* < .05 was considered statistically significant.

## RESULTS

### AlphaMissense Classifies a High Frequency of Missense Mutations in DDR Genes as Pathogenic

**FIG 1.**
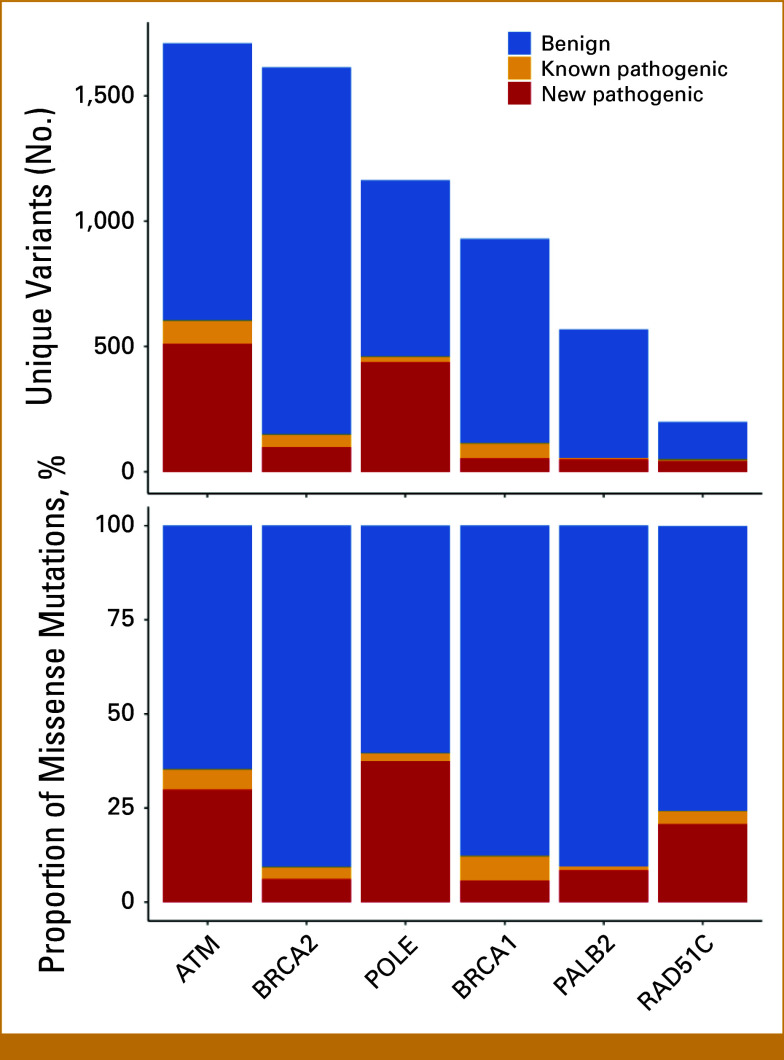
Frequency of missense mutations in DDR genes. Frequency of missense mutations in selected DDR genes identified in MSK-IMPACT cohort stratified according to pathogenicity classification. DDR, DNA damage repair; MSK-IMPACT, Memorial Sloan Kettering–Integrated Mutation Profiling of Actionable Cancer Targets.

In the MSK-IMPACT cohort, we identified 10,305 missense mutations (6,743 unique) in six selected DDR genes (Data Supplement, Table S1). AlphaMissense predicted 1,331 (19.7%) unique missense mutations as likely pathogenic. Applying our categorization (see Methods), 148 (2.2%) mutations were known pathogenic, 1,182 (17.5%) were new pathogenic, and 4,757 (70.5%) were benign missense mutations. Proportions of new pathogenic mutations varied by gene and were more frequent in *ATM* and *POLE* than in *BRCA1* and *BRCA2* (Fig 1). Pathogenicity prediction by OncoKB and AlphaMissense in *BRCA1* and *BRCA2* showed high overall agreement (93.3% in *BRCA1*, 92.8% in *BRCA2*), but lower in *ATM* (67.8%) and *POLE* (63.4%; Data Supplement, Fig S2).

### Newly Identified Pathogenic Missense Mutations in HR Genes Are Characterized by Classical Mutational Signatures of HR Deficiency

**FIG 2.**
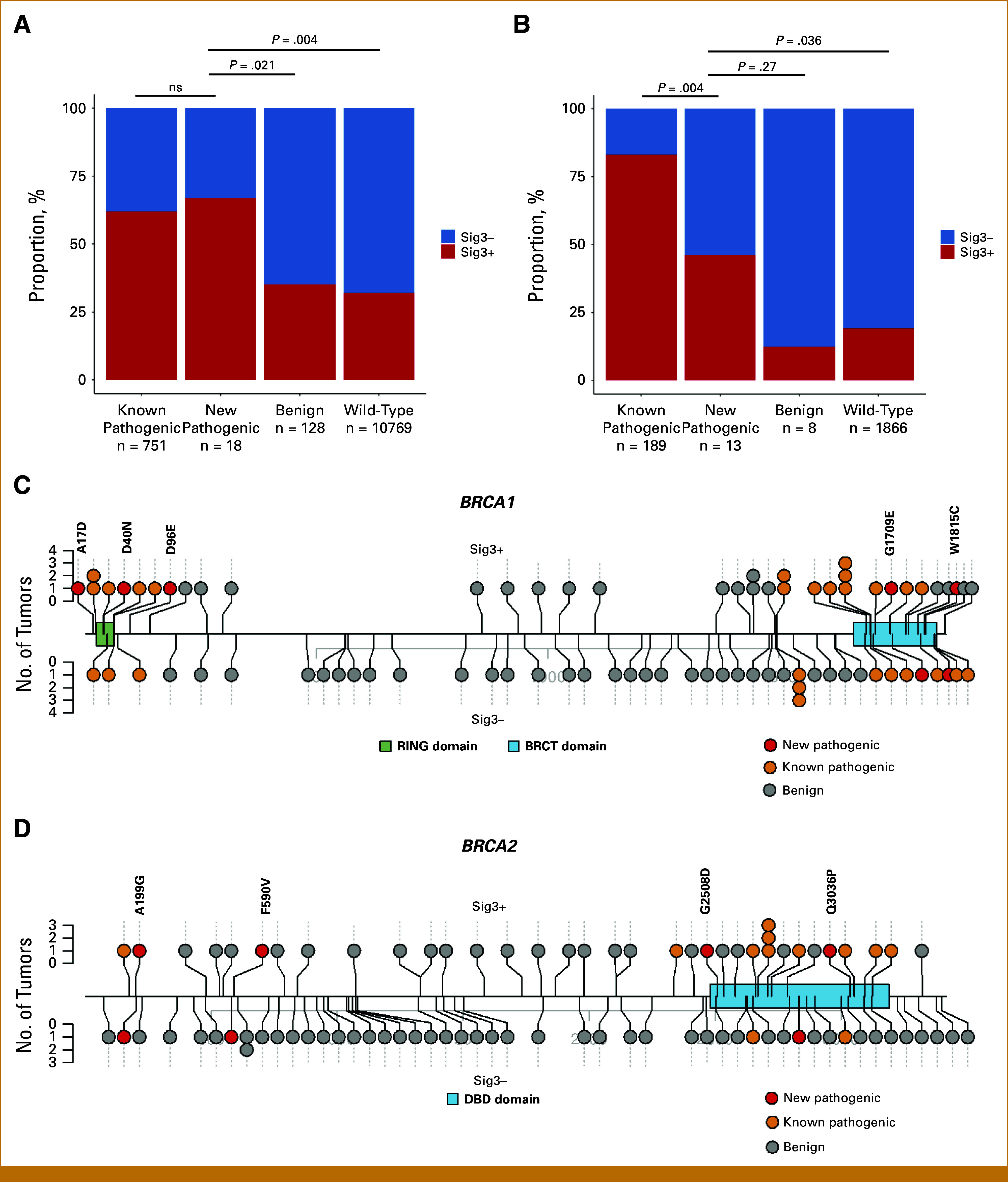
Genomic characteristics in tumors with missense mutations in core-HR genes. Association of signature three positivity and biallelic mutations in core-HR genes (*BRCA1/2*, *PALB2*, and *RAD51C*) in BOPP tumors in (A) the MSK-IMPACT cohort and (B) the TCGA/PCAWG cohort (*P* values calculated using the chi-square test; ns, nonsignificant, ≥.05). Biallelic missense mutation locations in (C) *BRCA1* and (D) *BRCA2* in the MSK-IMPACT cohorts stratified by signature three positivity (top Sig3-positive; bottom Sig3-negative). BOPP, breast, ovary, pancreatic, and prostate; BRCT, BRCA1 C terminus; DBD, DNA-binding domain, HR, homologous recombination; MSK-IMPACT, Memorial Sloan Kettering–Integrated Mutation Profiling of Actionable Cancer Targets; TCGA/PCAWG, The Cancer Genome Atlas/Pan-Cancer Analysis of Whole Genomes.

As both preclinical and previous clinical analyses suggest that biallelic inactivation of HR genes is required for HR deficiency–associated mutational signatures,^25,26^ we excluded monoallelic mutations in subsequent analyses. In MSK-IMPACT cohort, biallelic mutations in core-HR genes were identified in 897 (7.5%) of 11,666 patients with BOPP cancer, of which 51% were germline mutations (Data Supplement, Table S2). Among 187 patients with biallelic missense mutations, 18 (9.6%) had newly identified pathogenic mutations, including 14 in *BRCA1/2*, three in *RAD51C*, and one in *PALB2* (Data Supplement, Table S3). Sig3 positivity was higher in tumors with newly identified pathogenic missense mutations in core-HR genes than in benign mutations (66.7% *v* 35.2%, *P* = .021), while not different from known pathogenic mutations (66.7% *v* 61.9%, *P* = .87, Fig 2A). Absolute Sig3 exposure in tumors with new pathogenic missense mutations was also higher than in benign tumors (0.36 [0-0.62] *v* 0.02 [0-0.29], *P* = .033) and was not different from known pathogenic mutations (0.36 [0-0.62] *v* 0.32 [0-0.55], *P* = .84, Data Supplement, Fig S3A). New pathogenic *BRCA1/2* missense mutations, like known pathogenic missense mutations, were mainly located within previously described functional domains (Figs 2C and 2D). In TCGA/PCAWG cohort, biallelic new pathogenic missense mutations in core-HR genes were identified in 13 (0.6%, WES n = 12, WGS n = 1) of 2,076 patients with BOPP cancers. Because of the limited number of benign mutations (n = 8), we used wild-type tumors as a negative control. Tumors from TCGA/PCAWG with new pathogenic core-HR missense mutations had higher rates of Sig3 positivity (46.2% *v* 19.2%, *P* = .036, Fig 2B) and Sig3 exposure (0.20 [0-0.42] *v* 0 [0-0.16], *P* = .01, Data Supplement, Fig S3B) than in wild-type tumors, but significantly lower Sig3 positivity (46.2% *v* 83.1%, *P* = .004, Figs 2B) and Sig3 exposure (0.20 [0-0.42] *v* 0.39 [0.26-0.49], *P* = .02, Data Supplement, Fig S3B) compared with tumors with known pathogenic mutations. We further found additional anecdotal evidence that newly classified pathogenic variants were functional. Of the sole tumor with a new pathogenic variant and WGS, we used a more robust HRD signature method, HRDetect,^17^ and identified it had a high score (>0.99). This variant (p.Q2829H in *BRCA2*) has been reported to cause alternative splicing by in silico prediction and transcriptomic analysis.^27^ Furthermore, a review of medical records of MSK-IMPACT cases identified that three of 18 patients with new pathogenic core-HR gene mutations received PARP inhibitors as maintenance treatment. This included a patient with stage IIIC high-grade serous ovarian cancer with germline *BRCA1* p.D96E mutation and Sig3 positivity who benefited from treatment with a PARP inhibitor. After response to first-line platinum-based chemotherapy, she received PARP inhibitor as maintenance treatment and showed no evidence of recurrence 5 years after treatment, consistent with the therapeutic effect on deleterious *BRCA1/2* mutations.^28^ This variant remains a VUS in the ClinVar and OncoKB databases, although functional analysis supports that it causes a LoF.^29^

### AlphaMissense Does Not Reliably Identify Functional Mutations in *POLE*

**FIG 3.**
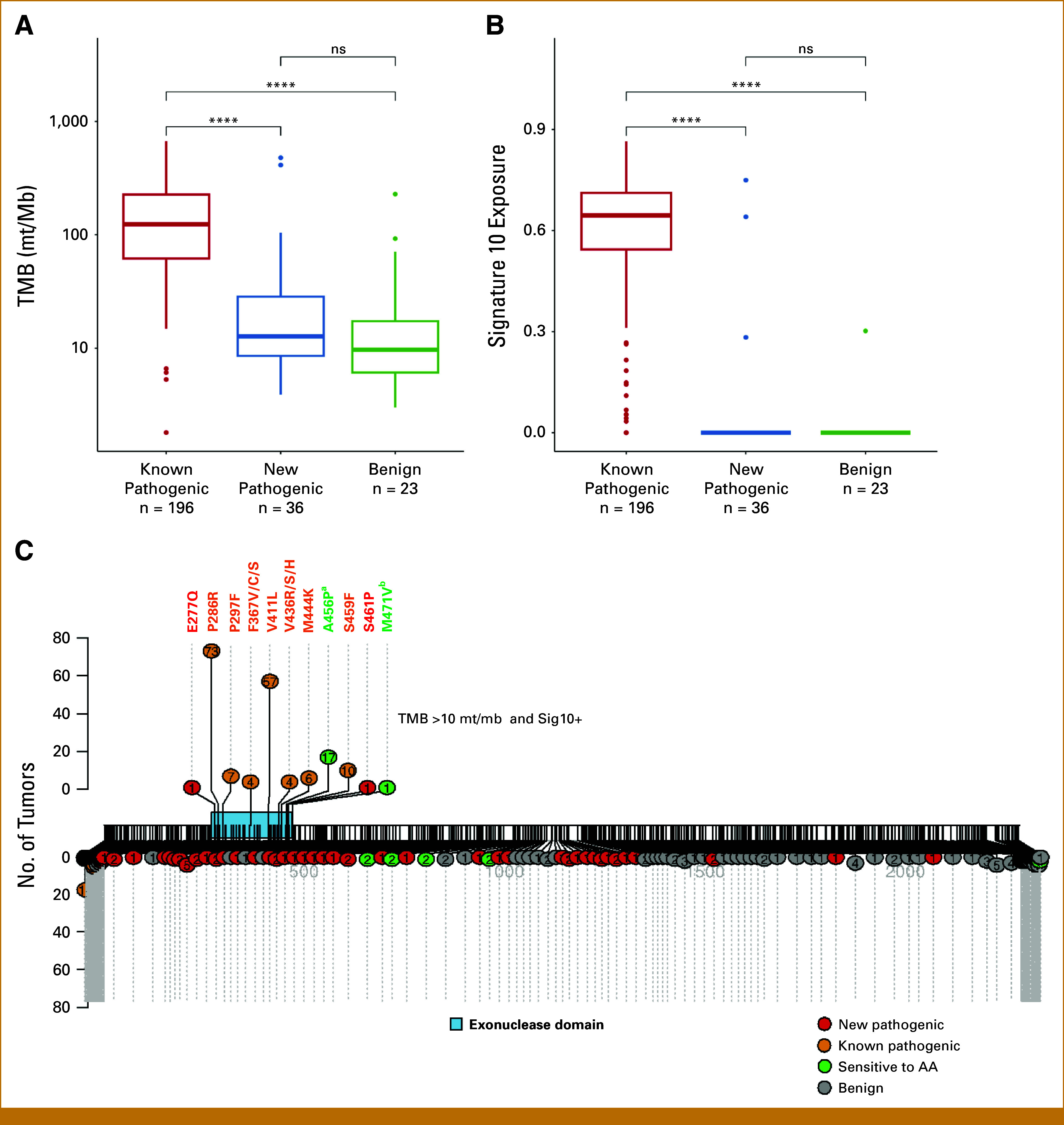
Genomic characteristics in tumors with *POLE* missense mutations. Association of (A) TMB, (B) POLE signature, and *POLE* ED missense mutations stratified by pathogenicity (*P* values calculated using the Wilcoxon signed rank test; ns, nonsignificant, ≥.05; ****, <.0001). (C) Mutation sites in *POLE* missense mutations in the MSK-IMPACT cohort. ^a^A456P was known pathogenic and A456T was new pathogenic; ^b^M471V was benign and M471I was new pathogenic. AA, amino acid; ED, exonuclease domain; MSK-IMPACT, Memorial Sloan Kettering–Integrated Mutation Profiling of Actionable Cancer Targets; TMB, tumor mutation burden.

Pathogenic mutations in *POLE* are known to lead to a hypermutator phenotype, with a characteristic mutational signature (Signature 10) that can make tumors with these mutations exquisitely sensitive to immunotherapy. The majority of pathogenic mutations are known to be in the ED, which is associated with a proofreading defect. In 937 patients with *POLE* missense mutations in the MSK-IMPACT cohort, we identified 279 (29.8%) with newly identified pathogenic *POLE* missense mutations, including 36 with ED and 243 with non-ED mutations (Data Supplement, Tables S4 and S5). TMB and POLE signature exposure showed no difference between tumors with new pathogenic and benign missense ED mutations (TMB, 12.8 [8.6-28.8] *v* 9.7 [6.1-17.6], *P* = .31; POLE Sig, 0 [0-0.75] *v* 0 [0-0.30], *P* = .56). New pathogenic ED mutations had lower TMB and POLE signatures than those of known pathogenic ED mutations (TMB, 12.8 [8.6-28.8] *v* 124 [62-226], *P* < .001; POLE Sig, 0 [0-0.75] *v* 0.64 [0.54-0.71], *P* < .001; Figs 3A and 3B). There was no difference in TMB and POLE signatures between new and known pathogenic non-ED mutations (TMB, 13.2 [7-31.1] *v* 26.2 [10.4-38.6], *P* = .23; POLE Sig, 0 [0-0.07] *v* 0 [0-0], *P* = .71; Data Supplement, Figs S4A and S4B). Of 279 patients with newly identified pathogenic missense mutations, only two (0.7%) patients with ED mutations (E277Q, S461P) showed POLE signature–positive and TMB-high (E277Q: 411.6 mut/Mb, S461P: 103.8 mut/Mb), suggesting that these *POLE* mutations maybe true drivers (Fig 3C). One of the two patients was treated with ICB. A patient with recurrent urothelial carcinoma of the bladder with *POLE* p.E277Q mutation achieved a complete response with an anti–PD-1 antibody and had no disease progression for over 3 years (Data Supplement, Fig S5).

### Genomic and Clinical Features of New Pathogenic *ATM* Missense Mutations

**FIG 4.**
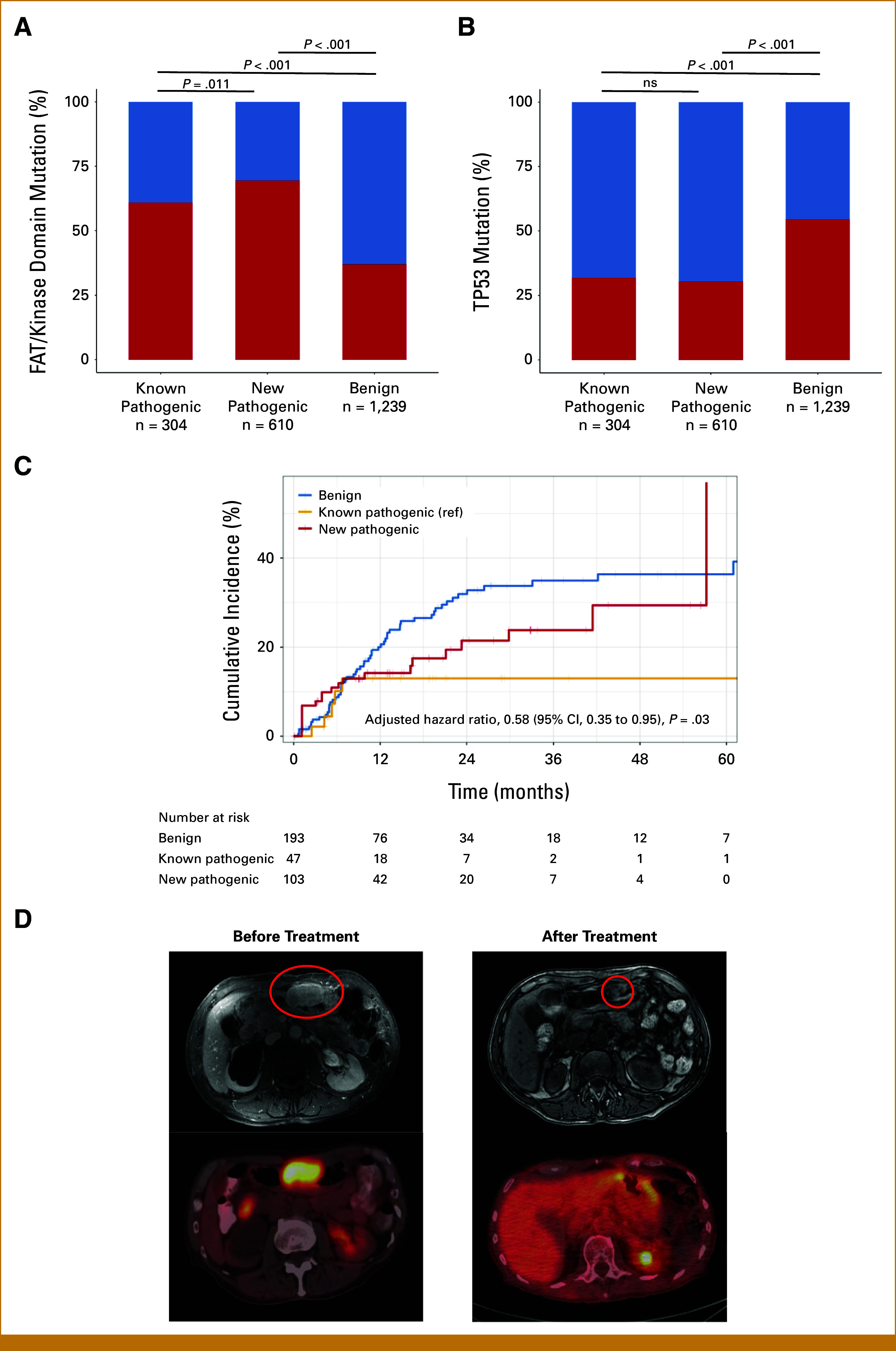
Genomic and clinical characteristics in tumors with *ATM* missense mutations. Frequency of (A) FAT/kinase domain mutations and (B) co-occurrent *TP53* mutations in known pathogenic versus new pathogenic versus benign *ATM* missense mutations in MSK-IMPACT cohort (*P* values calculated using the chi-square test; ns, nonsignificant, ≥.05). (C) Cumulative incidence of irradiated tumor progression by pathogenicity of *ATM* missense mutations. (D) Exceptional responder to RT in a patient with urothelial cancer with *ATM* new pathogenic mutation (p.E158K). Abdominal MRI and PET scan of gastric metastases showing near-complete response 7 months after RT. The red circles highlight a target lesion. FAT, FRAP, ATM and TRRAP; MRI, magnetic resonance imaging; MSK-IMPACT, Memorial Sloan Kettering–Integrated Mutation Profiling of Actionable Cancer Targets; PET, positron emission tomography; ref, reference; RT, radiotherapy.

Although *ATM* is a recurrently mutated DNA repair gene in cancer, it does not have a well-described mutational signature associated with it.^30^ Hence, to evaluate whether newly identified pathogenic mutations in AlphaMissense were functional, we relied on well-described genetic associations with *ATM* mutations (mutual exclusivity with *TP53* mutations) and previously described responses to RT.^11,30^ Among 2,153 patients with *ATM* missense mutations in the MSK-IMPACT cohort, 610 (28.3%) had newly identified pathogenic *ATM* mutations (Data Supplement, Table S6). Newly identified pathogenic mutations and known pathogenic mutations were more likely to be in the FAT/kinase domain than benign mutations (*P* < .001 for both); however, they were slightly more common in newly identified mutation cases (69.5% *v* 60.9%, *P* = .011; Fig 4A, Data Supplement, Fig S6). Both newly identified pathogenic mutations and known pathogenic mutations had lower rates of *TP53* comutation (*P* < .001 for both), and rates of TP53 comutations were not different between the two (30.5% *v* 31.9%, *P* = .72, Fig 4B). Next, we evaluated the association between *ATM* genotypes and irradiated tumor progression among 168 patients with *ATM* missense mutations who received RT to 343 lesions. Patients and treatment characteristics are provided in the Data Supplement (Table S7 and S8). We found that tumors with new pathogenic *ATM* missense mutations were significantly associated with improved irradiated tumor control than those with benign missense mutations, according to a multivariable model (hazard ratio, 0.58 [95% CI, 0.35 to 0.95]; *P* = .03, Fig 4C, Data Supplement, Table S9). A review of individual clinical histories highlighted exceptional responses to RT. This includes a patient with metastatic urothelial carcinoma in the lung and stomach harboring *ATM* p.E158K mutation. He experienced near-complete response to palliative RT for gastric bleeding and had no in-field recurrence for over 5 years (Fig 4D).

### AlphaFold Protein Structure Predictions of DDR Genes

Finally, we examined whether the accuracy of AlphaMissense prediction was associated with the accuracy of AlphaFold structure predictions. Most residues in the functional domain of *ATM*, *BRCA1*, *BRCA2*, and *POLE* have very high or high confidence levels for local structure (pLDDT ≥70), especially for *BRCA1* and *POLE* (Data Supplement, Fig S7). However, the similarity between AlphaFold and experimentally derived structure varied by gene, with the lowest for *POLE* (PDB ID: 7PFO; RMSD of atomic positions = 3.32 Å; Data Supplement, Fig S8).

## DISCUSSION

Here, we assessed the accuracy of AlphaMissense for predicting the pathogenicity of missense mutations in DDR genes, on the basis of genomic characteristics and therapeutic vulnerabilities. We showed that AlphaMissense identified previously unknown pathogenic missense mutations in DDR genes. However, the accuracy and clinical relevance differed across genes.

Newly identified pathogenic core-HR missense mutations were associated with HR-deficiency signatures, and 46%-67% of these mutations had orthogonal evidence that they were functionally pathogenic. However, only 10% of patients with missense mutations in core-HR genes were reclassified as pathogenic, indicating a limited number of patients who would be reclassified and amenable to new therapies. This may be attributed to the fact that *BRCA1/2* are the most well-studied DDR genes, and which already have extensive functional evaluation of many missense mutations, as shown by the high concordance between OncoKB and AlphaMissense annotations. According to a study that performed a functional analysis of single-nucleotide variants in the *BRCA2* DBD domain using the saturation genome editing approach, missense mutations predicted to be pathogenic were associated with a higher frequency of LOH and an increased risk of breast and ovarian cancers.^31^ However, similar to the current study, only about 4% of the ClinVar VUS were reclassified as pathogenic, while most were reclassified as benign.

By contrast, new pathogenic *POLE* mutations were not associated with high TMB and POLE-proofreading defect signatures. We found that <1% of newly identified pathogenic *POLE* mutations had characteristic genomic features. One of them achieved a complete response to ICB treatment. A previous study showed that 38% of patients with *POLE* VUS mutations had an objective response to ICB (48% with pathogenic, 0% with benign), suggesting that current annotation of VUS may not accurately predict benefits of ICB.^32^ Our previous study demonstrated that patients with *POLE* mutations harboring function-associated signatures responded better to ICB than those without such signatures. Furthermore, a signature-based approach outperformed other traditional approaches, such as hypermutation and ED mutation, in identifying *POLE*-mutated tumors likely to benefit from ICB benefit.^33^ These findings underscore the need for improved functional prediction of VUS to better understand differences in immunotherapy response.

We found that newly identified pathogenic *ATM* mutations were mutually exclusive with *TP53* mutations and showed radiosensitivity. Patients with germline or somatic *ATM* mutations are hypersensitive to ionizing radiation. Indeed, germline carriers of homozygous *ATM* mutations cause ataxia-telangiectasia, a human genetic disorder associated with the highest radiosensitivity. Our group previously identified that *ATM* mutations in tumors are exquisitely sensitive to ionizing radiation in in vitro studies^34^ and were associated with clinical benefit from RT in large cancer cohorts.^11^ Given these characteristics, we relied on radiation sensitivity to assess the functional accuracy of AlphaMissense prediction. Missense mutations in *ATM* were the most common among DDR genes and had the highest number of newly identified pathogenic mutations, suggesting that AlphaMissense predictions may have a significant impact on clinical decision making. Recent clinical studies showed that patients with LoF mutations in *ATM* could benefit from DDR-targeted therapies such as ATR inhibitors as well as RT,^35,36^ underscoring the importance of identifying *ATM* missense mutations with functional impacts for future treatment strategies.

To understand the mechanism behind this gene-specific difference, we examined differences in the accuracy of AlphaFold protein structure prediction. However, the confidence levels of local structures were similarly high across genes. One possible explanation is that AlphaMissense is better suited for predicting complete LoF mutations, which result in substantial disruption of protein structure or function, than for predicting partial LoF or gain-of-function mutations. *POLE* mutations associated with cancer susceptibility and hypermutation do not cause complete LoF; instead, they result in the selective loss of exonuclease (proofreading) activity while retaining polymerase function. This domain-specific alteration may complicate accurate pathogenicity prediction in the context of POLE-associated tumors.

This study has several limitations. First, we relied on Sig3 as a marker of HR deficiency, and other genomic markers, including deletions with microhomology and rearrangement signatures,^17^ could not be examined from patients with MSK-IMPACT targeted sequencing data. In addition, targeted panel sequencing is not the best method to detect mutational signatures.^37^ Compared with the TCGA/PCAWG data set, the MSK-IMPACT cohort has a higher proportion of Sig3 positives in wild-type tumors, which likely include some false-positive results. Sig3 is a relatively flat and broad mutation distribution without strong peaks at specific trinucleotide contexts, making it inherently difficult to distinguish from signatures such as SBS1 and SBS5, especially when there are a limited number of mutations to analyze. Although it has limitations, multiple studies have shown, even in targeted sequencing data, Sig3 correlates strongly with HR deficiency.^20,38^ Second, the small population of patients with new pathogenic core-HR gene or *POLE* mutations and who received targeted therapy precluded meaningful investigation of clinical outcomes after targeted therapy.

In conclusion, AlphaMissense contributed to identifying true pathogenic missense mutations in DDR genes, while its accuracy varied by genes. The pathogenicity prediction by AlphaMissense should be validated with clinical and functional evidence before being applied to clinical decision making.

## Data Availability

The data analyzed in this study are available from the corresponding author upon reasonable request.
